# Hepatitis C Virus NS3/4A Protease Inhibitors: A Light at the End of the Tunnel

**DOI:** 10.3390/v2081752

**Published:** 2010-08-20

**Authors:** Laurent Chatel-Chaix, Martin Baril, Daniel Lamarre

**Affiliations:** Institut de Recherche en Immunologie et en Cancérologie (IRIC), Montréal, Québec, H3T 1J4, Canada; E-Mails: laurent.chatel.chaix@umontreal.ca (L.C.-C.); martin.baril@umontreal.ca (M.B.)

**Keywords:** HCV, protease inhibitor, NS3 protease, antiviral therapy, HCV replicon, clinical trial

## Abstract

Hepatitis C virus (HCV) infection is a serious and growing threat to human health. The current treatment provides limited efficacy and is poorly tolerated, highlighting the urgent medical need for novel therapeutics. The membrane-targeted NS3 protein in complex with the NS4A comprises a serine protease domain (NS3/4A protease) that is essential for viral polyprotein maturation and contributes to the evasion of the host innate antiviral immunity by HCV. Therefore, the NS3/4A protease represents an attractive target for drug discovery, which is tied in with the challenge to develop selective small-molecule inhibitors. A rational drug design approach, based on the discovery of N-terminus product inhibition, led to the identification of potent and orally bioavailable NS3 inhibitors that target the highly conserved protease active site. This review summarizes the NS3 protease inhibitors currently challenged in clinical trials as one of the most promising antiviral drug class, and possibly among the first anti-HCV agents to be approved for the treatment of HCV infection.

## 1. Introduction

Chronic Hepatitis C virus (HCV) infection is a serious cause of liver disease worldwide that leads to progressive fibrosis, and may result in cirrhosis, hepatocellular carcinoma, liver failure and death. Chronicity is established through a very high genetic variability of HCV together with several powerful strategies of the virus to evade host immunity. The HCV NS3/4A protein is a membrane-targeted serine protease responsible for maturation of the viral polyprotein, which cleaves four non structural (NS) sites to generate the mature NS3, NS4A, NS4B, NS5A and NS5B proteins. The NS3 protease activity is essential for viral replication, as was demonstrated directly by the non-productive infection following liver inoculation of an active site mutated HCV molecular clone in chimpanzees [1]. As viruses developed a myriad of host immune evasion strategies, a central role of the NS3/4A protease was established following the elegant elucidation of mechanisms used by HCV to counteract antiviral innate immune signaling. The NS3/4A protease interferes with the pathogen recognition TLR3- and RIG-I/MDA5-mediated signaling pathways, by cleaving TRIF and MAVS (also known as Cardif, VISA or IPS-1) signaling adaptors, respectively, preventing the transcriptional activation of type I interferon (IFN) genes and IFN-stimulated genes (ISGs) [2]. For these reasons, the NS3/4A protease is an intensively studied viral protein that represents one of the most attractive targets for drug discovery.

## 2. Need for improved HCV therapeutic regiments using combination strategies

The standard of care (SOC) for HCV infected patients consists of a weekly administration of pegylated interferon alpha combined with a twice-a-day dose of ribavirin (Peg-IFN/Rib). The treatment is 48-weeks long for HCV genotype 1 infected patients and 24–weeks long for genotypes 2 and 3. The therapeutic benefit is determined by the sustained viral response (SVR) and is achieved when the levels of plasma HCV RNA are below the detection limit at 24 weeks after the end of the treatment. The rate of SVR is approximately 50% for genotype 1 and 80% for genotype 2 and 3 [3]. The current therapies provide limited efficacy and are poorly tolerated, a consequence of combining two nonspecific antiviral agents with pleiotropic effects that were not originally designed to treat chronic HCV infection. The undefined nature of the current treatment makes it very difficult to pharmacologically refine combination strategies, especially in the treatment of a significant proportion of patients that have experienced drug resistance to SOC and therapeutic failure. This situation highlights the need for well-defined antiviral and immunomodulatory therapeutics to rationalize mechanism-based combination strategies in treatments achieving a high genetic barrier to resistance and restoring HCV-specific immunity. Hence, specific therapeutic strategies have to be elaborated in order to cure HCV infection. Target-based antiviral drug discovery that mainly relies on the use of *in vitro* assays, has led to the identification of several anti-HCV compounds awaiting clinical validation through tangible therapeutic benefit in HCV-infected patients.

## 3. Design of NS3 protease inhibitor BILN 2061: First anti-HCV proof-of-concept in man

**Figure 1.**
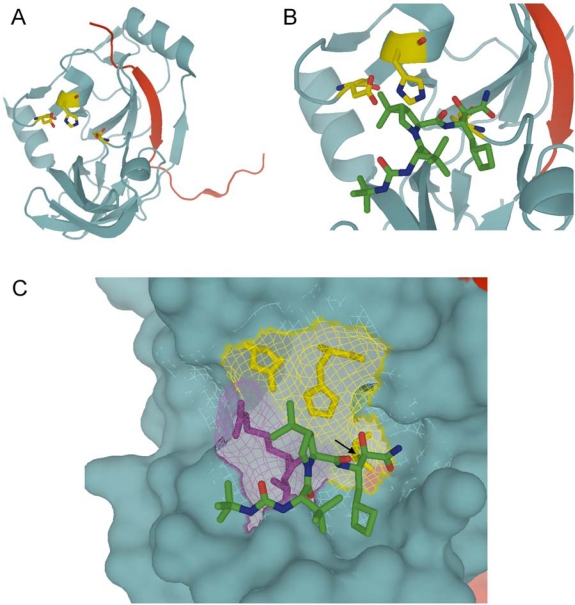
Representations of the boceprevir bound to the NS3/4A protease domain. **(A)** Ribbon drawing of the tertiary structure of a monomer NS3/4A protease domain. The NS4A peptide is shown in red. The side chains of the catalytic triad (H57, D81 and S139) are shown as yellow ball-and-stick models. **(B)** Zoomed-in view of the NS3/4A active site with the boceprevir represented as sticks in atom specific coloring (green for carbon, red for oxygen, and blue for nitrogen). **(C)** The bulk of the protein is shown as a Connolly surface, while residues of the catalytic triad (yellow) and residues R155 and A156 for which mutations confer resistance to most NS3 protease inhibitors (purple) are represented as mesh surface with the position of the side chains shown as sticks. The arrow points to the reversible covalent bond formed between the boceprevir and the active site S139. This figure was generated with PDB ID number: 2OC8 [31] using Pymol.

Since the probability of SVR achievement positively correlates with the rapid and significant reduction of plasma HCV RNA, combination of anti-HCV candidate drugs achieving sustained antiviral suppression with possible immunotherapy should aim at eradicating infection in all patients. Hence, many efforts have been made to identify molecules that directly and specifically target essential viral functions (DAA: Direct-Acting Antiviral). With the insights gained in the design of human immunodeficiency virus (HIV) protease inhibitors for the treatment of AIDS, and the discovery of N-terminus product inhibitors of NS3 protease, rational drug design approaches were undertaken to develop selective HCV inhibitors with promise in blocking viral replication in infected patients. Despite retaining some genetically conserved features of the chemotrypsin serine protease family (such as the spatial organization of the catalytic triad), NS3 X-ray structure revealed a substrate binding groove that is shallow and relatively exposed to solvent as compared to others serine proteases (Figure 1A) [4,5]. Because of this unique topography, the design of NS3 active site inhibitors represented a big challenge. To date, all developed NS3/4A inhibitors in clinical trials are peptide-based compounds derived from cleavage products, and hence target the serine protease active site (Table 1).

Ciluprevir or BILN 2061, discovered at Boehringer Ingelheim in Canada, was the first-in-class NS3 protease inhibitor compound ever tested in human for the treatment of HCV infection. Pre-clinical data indicated that BILN 2061 is a non-covalent specific and potent competitive inhibitor of the NS3/4A protease genotype 1, and a potent inhibitor of HCV RNA replication that blocks HCV polyprotein processing, consistent with its designed mode of action. From *in vitro* studies, MAVS cleavage by NS3 protease in HCV-infected Huh7 cells in culture is completely abrogated by BILN 2061 treatment, demonstrating a dual therapeutic potential of protease inhibitors to restore antiviral innate signaling [6]. When orally administered to chronically infected patients, ciluprevir induced a 2–4 log_10_ IU/mL decline in plasma HCV RNA in two days [7]. These very promising results represented the first clinical proof-of-concept of DAA efficiency *in vivo*. Although ciluprevir development was halted in phase Ib clinical trial because of toxicity in animals, the clinical results prompted the development of other NS3/4A inhibitors. Notably, ciluprevir scaffold was exploited to design new macrocyclic inhibitors such as TMC-435, danoprevir and others (Table 1).

## 4. NS3 protease inhibitors in clinical development

### 4.1. Telaprevir

Telaprevir or VX-950 (Vertex Pharmaceuticals Inc., MA, and Tibotec BVBA, Belgium) is a linear peptidomimetic NS3/4A inhibitor that possesses an α-ketoamide group serine trap warhead forming a covalent but reversible complex with a steady-state inhibition constant (Ki) of 7 nM against the enzyme. The Ki is 4-7-fold and 40-fold higher for genotype 2 and 3, respectively, suggesting that its potential therapeutic use would need genotype-optimization (see below). VX-950 demonstrated antiviral activity *in vitro* with sub-micromolar inhibition of HCV genotype 1 RNA replication.

In phase IIa clinical trials conducted with treatment-naïve genotype 1 HCV-infected patients, telaprevir showed a marked reduction in the viral load of patients (1.3–5.3 log_10_ IU/mL) in monotherapy for 15 days at a dose of 750 mg every 8 hours. The phase II PROVE (protease inhibitor for viral eradication)-1 and -2 trials consisted of a 12-week lead-in with Peg-IFN/Rib/telaprevir triple therapy regimen followed by 36 (PROVE-1) or 12 (PROVE-2) weeks of Peg-IFN/Rib treatment [8,9]. All telaprevir arms showed an increase in SVR achievement to 67% and 69% as compared to 41% and 46% for SOC for PROVE-1 and -2, respectively. These results suggest that Peg-IFN/Rib treatment duration could be shortened and hence adverse effects possibly attenuated.

PROVE-3 consisted of the same treatment strategy in patients that previously failed SOC regimen. The SVR rate of previous SOC non-responders was 38–39% for patients who received Peg-IFN/Rib/telaprevir triple therapy as compared to 9% for those re-treated with SOC only. Additionally, the SVR rate for prior relapsers was 69–76% *versus* 20% for SOC [10]. These encouraging results suggest that telaprevir-containing triple therapy regimen can maximize SVR rates in patients who previously failed SOC [11]. Notably, these studies showed that ribavirin reduces virological breakthrough and contributes to the optimal efficacy of the triple therapy.

Unfortunately, the high replication rate of HCV combined with the low fidelity of the viral polymerase NS5B led to the emergence of drug resistance mutations in NS3 within two weeks of telaprevir monotherapy (see Table 1). These NS3 mutations observed in patients conferred low-level resistance (<25-fold increase in IC_50_): V36M/A, T54A, R155K/T, A156S or high-level resistance (>50-fold increase in IC_50_): A156V/T, V36M/A + R155K/T, V36M/A + A156V/T; and were all located in or close to the inhibitor binding site and to the catalytic site of NS3 protease. In addition to an increased antiviral activity, co-administration of Peg-IFN/Rib with telaprevir greatly reduced the incidence of viral resistance. The clinical data support that an efficient antiviral treatment must combine multiple antiviral drugs independently targeting HCV with no overlapping resistance profile.

As compared to SOC, telaprevir was associated with no additional adverse effects, but increased rates of certain symptoms including gastrointestinal events, anemia and severe skin rash. Notably, the latter was a major cause of triple therapy discontinuation, even if this symptom resolved following the end of Peg-IFN/Rib/telaprevir treatment.

All these studies show that telaprevir represents a promising new anti-HCV drug. Telaprevir is now being evaluated in phase III of clinical development within three trials: ADVANCE, ILLUMINATE and REALIZE, in order to ascertain the critical determinants of a triple therapy-based strategy for optimal SVR achievement. Very recently, the analysis of ADVANCE trials revealed that adding telaprevir to the regimen during the first 8 or 12 weeks of Peg-IFN/Rib treatment significantly improved the SVR rate from 44% for the SOC control arm to 69% and 75%, respectively, in treatment-naïve patients [12]. These results are in line with results previously obtained in phase II trials. Notably, improvements in the treatment discontinuation rate due to adverse effects were noticed. While ILLUMINATE and REALIZE first results will be available in the third quarter of 2010, Vertex is preparing for a possible commercial launch of telaprevir in early 2011.

### 4.2. Boceprevir

Boceprevir or SCH 503034 (Merck & Co., NJ; initially developed by Schering-Plough) is a linear peptidomimetic NS3/4A protease inhibitor possessing a serine trap (Figure 1B and 1C) that reversibly forms a covalent bond with the enzyme (Ki of 14 nM). After 14 days of boceprevir monotherapy in patients who did not achieve SVR after SOC, plasma HCV RNA levels were decreased by ∼1.6 log_10_ IU/mL. Co-treatment with Peg-IFN increased this antiviral effect to 2.9 log_10_ IU/mL.

After these promising results, Schering-Plough initiated the phase II clinical trial SPRINT-1 (serine protease inhibitory therapy) that consisted of a four week lead-in with Peg-IFN/Rib followed by a 44 or 24 week Peg-IFN/Rib/boceprevir triple therapy regimen in treatment-naïve patients infected with genotype 1 HCV [13]. Astoundingly, results of these studies showed that the triple therapy arm was associated with a significant increase of SVR rate (75% and 56% for 44 and 24 weeks triple therapy, respectively) as compared to the SOC arm (38%). Importantly, an SVR rate of 82% was achieved in patients who had a rapid viral response (RVR: HCV RNA below the limit of detection at week 4) following the four week lead-in, and were further treated for 24 weeks with the boceprevir-based regimen. Hence, as for telaprevir, these studies showed that reducing treatment duration from 48 to 28 weeks might be achievable without decreasing the SVR rate. Boceprevir represents a promising novel anti-HCV drug and has now entered in phase III of clinical development with the SPRINT-2 trial.

Another phase II clinical trial, RESPOND-1, evaluated the therapeutic potential of a similar strategy in patients who previously failed SOC. Results for this trial were disappointing as only 7–14% of patients achieved SVR [14]. This is being currently reevaluated in the phase III RESPOND-2 trial.

Similarly to with telaprevir treatment, several drug resistance mutations (see Table 1) emerged in the NS3 protease upon boceprevir treatment. An overlapping resistance profile is observed for boceprevir and telaprevir, suggesting that the combination of these two drugs in a putative therapy will not increase the selective pressure on HCV and hence, does not represent a promising therapeutic avenue.

Fatigue, anemia, nausea, dysgeusia and headache were reported as adverse effects in boceprevir-treated patients. However, the increased rate of anemia (44–48% in the boceprevir arms *vs.* 33% in the group without boceprevir) and dysgeusia were proportional to the SVR rate.

### 4.3. TMC 435

TMC 435 (TMC 435350) is a non-covalent macrocyclic NS3/4A protease inhibitor developed by Tibotec (Belgium) and Medivir (Sweden) who initiated the clinical trial OPERA-1, a double-blind, placebo-controlled phase II trial assessing TMC-435 antiviral activity [15]. Naïve, prior relapser and prior non-responder patients were given several daily doses of TMC 435 (75, 150 or 200 mg) in combination with Peg-IFN/Rib for four weeks. The triple therapy regimen is currently followed by a 44-weeks of Peg-IFN/Rib administration. Interim results from this study after 28 days revealed that 89% of the patients achieved RVR and that viral load decreased (4.7–5.4 log_10_ *vs.* 3.6 log_10_ for SOC only) in all TMC-435 arms, confirming the potent antiviral activity of TMC 435 in naïve and treatment-experienced patients. Observed adverse effects (nausea, diarrhea and headache) were not serious and never led to treatment discontinuation.

### 4.4. Danoprevir

Danoprevir or RG7227/ITMN-191 is a non-covalent macrocyclic inhibitor of HCV NS3/4A protease developed by Intermune Inc, CA, and Roche, NJ. Phase 1b studies showed up to 3.9 log_10_ viral load decrease with a 14-day danoprevir monotherapy in treatment-naïve genotype 1 patients. Further decrease in viral load was achieved (4.7–5.7 log10 IU/mL) when Peg-IFN/Rib was co-administered with danoprevir during the two weeks of treatment. Under the triple therapy regimen, 13–57% of patients showed undetectable plasma HCV RNA, compared with 0% under SOC [16]. Danoprevir-related adverse effects were qualified as “mild and transient” in initial phase I trial. Danoprevir is currently in Phase IIb of clinical development and interim analyses revealed that, following a 12 week tri-therapy, 88–92% of the patients achieved early viral response (*versus* 43% for the SOC control group) [17].

Notably, the CYP3A inhibitor, ritonavir, was shown to “boost” danoprevir pharmacokinetics. Consistently, ritonavir/danoprevir/SOC regimens provide more robust virological responses at lower danoprevir doses than unboosted regimens after 14 days of treatment.

Very interestingly, the combination of danoprevir with NS5B polymerase inhibitor RG7128 (PSI-6130; Roche) significantly increased antiviral activity in HCV replicon-containing cells *in vitro*. The therapeutic potential of the bi-therapy is being evaluated in the INFORM-1 clinical trial [18]. Interim results revealed increased and sustained antiviral effects of the RG7128/danoprevir combination *versus* monotherapy after two weeks. The adverse effects were acceptable and no treatment-emergent resistance was observed. The patients treated for 13 days with the highest dose of RG7128/danoprevir regimens followed by SOC for 12 weeks achieved RVR and early viral response (EVR: HCV RNA below the limit of detection at week 12) rates of 88% and 100%, respectively [19]. This antiviral strategy represents the first DAA-based combination therapy and is very promising.

### 4.5. Other NS3 protease inhibitors

Presently, numerous pharmaceutical companies have NS3 protease inhibitors in their pipeline, although limited information is generally available. Three inhibitors are currently in phase II clinical trials: vaniprevir (Merck & Co., NJ), narlaprevir (Merck & Co., NJ) and BI 201335 (Boehringer Ingelheim Pharma, Canada). When vaniprevir (MK-7009) was administered for four weeks as part of a triple therapy with Peg-IFN/Rib, the RVR rate was 69–82%, notably higher than the 5.6% observed with SOC. An additional eight weeks of SOC led to an EVR rate of 77–89% [20]. Narlaprevir (SCH 900518) in triple therapy led to a 4–4.5 log_10_ IU/mL viral load reduction after eight days in both treatment-experienced and naïve HCV genotype 1-infected patients. Preliminary results of the narlaprevir phase II trial (NEXT-1) showed that patients receiving a four week SOC lead-in followed by a triple therapy regimen had RVR and EVR rates of 58–87% and 84–87%, respectively [21]. A phase II trial is currently evaluating SVR rate with and without co-administration of ritonavir as a pharmacokinetic booster. Fourteen days of BI 201335 monotherapy resulted in a median HCV RNA decline of 3–4.2 log_10_ IU/mL, while an additional 14 days of triple therapy increased this decline to 4.8–5.3 log_10_ IU/ml [22]. Interim results from phase II SILEN-C1 trials showed that infected patients who received BI 201335/Peg-IFN/Rib triple therapy for 12 weeks had RVR and EVR rates of 92% and 91%, respectively, as compared to 16% and 42% in the control arm. This regimen also showed robust antiviral activity after 12 weeks in patients who did not previously respond to SOC as revealed by the SILEN-C2 study [23].

Other protease inhibitors currently in phase I of clinical development include VX-813, VX-500 and VX-985 (Vertex Pharmaceuticals Inc., MA), VBY-376 (ViroBay, CA), PHX1766 (Phenomix, CA), ABT-450 (Abbott, IL and Enanta Pharmaceuticals, MA), BMS-650032 (Bristol-Myers Squibb, NY), ACH-1625 (Achillion Pharmaceuticals, CT), MK-5172 (Merck & Co., NJ) and GS-9256 (Gilead Sciences, CA). Results of long-term treatments with these compounds are still unavailable.

## 5. Challenges and future directions

In order to maximize the efficacy of therapy and broaden the treatable population, the HCV protease inhibitors will have to be tested and studied in chronically infected patients who failed the SOC and also in populations that classically show low response rates (African-Americans, HIV-HCV co-infected individuals, patients with cirrhosis, kidney failure or liver transplant). In addition, the antiviral activity of these treatments will have to be evaluated in individuals infected with non-genotype 1 HCV. For instance, the study C209 revealed that telaprevir shows little or no antiviral activity against genotype 3 HCV while efficiently targeting both genotype 1 and 2 HCV [24]. This illustrates the importance of developing DAAs specifically targeting different genotypes or effective against multiple genotypes.

One of the biggest concerns about the development of an efficient anti-HCV therapy is to maximize the tolerability of the patient towards the treatment. Indeed, SOC is associated with severe adverse effects whose rate is further increased with DAAs presently in development. The administration of ribavirin is one of the major causes of adverse symptoms. However, removal of ribavirin from the antiviral cocktail significantly diminished the SVR rate obtained by treatment with telaprevir and boceprevir. It will be important to optimize new treatments that do not require the administration of ribavirin and eventually IFN-based therapy. Meanwhile, decreasing treatment duration from 48 to 24 weeks and administration frequency will be good alternatives for enhancing tolerability. Notably, the EVR rates are satisfactory in arms treated once daily with DAAs such as narlaprevir, MK-7009 and BI 201335.

A major challenge for a successful anti-HCV therapy is to delay the emergence of drug resistance and virological breakthrough that were reported in the first two weeks under DAA monotherapy. Interestingly, Merck has designed a novel NS3/4A inhibitor, MK-5172, which retains subnanomolar potency across genotypes and NS3 variants with key clinical resistance mutations [25]. This drug is undergoing phase I development and may exhibit *in vivo* a high barrier to the development of viral resistance. To limit virus resistance emergence, a strong selective pressure is required to reduce HCV replication and maintain long-term virus suppression as with antiviral combination therapy for HIV infection. Similarly, the solution for HCV will reside in a treatment that includes multiple DAAs with additive or synergistic antiviral potency, and more importantly with no overlapping resistance. Results from the trials highlighting the antiviral activity of danoprevir/RG7128-based regimens (see above) are very encouraging and represents the first proof of concept of this anti-HCV therapeutic strategy. More recently, several phase II clinical trials evaluating the antiviral activity of combination regimens based on telaprevir and VX-222 (an HCV polymerase inhibitor), GS-9256 and GS-9190 (an HCV polymerase inhibitor) or BMS-650032 and BMS-790052 (a NS5A inhibitor) with or without Peg-IFN/Rib were launched by Vertex, Gilead Sciences and Bristol-Myers Squibb, respectively. Hopefully, these treatments will alleviate the need for Peg-IFN/Rib and reduce the incidence and the gravity of adverse effects.

Another challenge resides in the identification of new classes of NS3/4A protease inhibitors. Achillion Pharmaceuticals (CT) is developing ACH-1095, an NS4A antagonist, which is essential for the NS3 serine protease activity. This compound is currently in late-stage preclinical assessment. Furthermore, inhibitors targeting the NS3 helicase domain could affect its protease activity given that the NS3 sub-domains mutually regulate each other [26–28]. Furthermore, the identification of conserved and critical NS3/4A protein-host factor interactions could lead to exciting therapeutic avenues by hopefully minimizing the incidence of resistance mutations.

Finally, the genotypic analysis of specific genes in HCV-infected patients will probably help to predict SVR achievement. Indeed, it has been recently demonstrated that polymorphisms in the IL28B gene region of chromosome 19 is strongly associated with SOC-induced clearance of HCV genotype 1 infection [29]. Very recently, Akuta *et al.* have shown that the likelihood of SVR following telaprevir/Peg-IFN/Rib triple therapy is also improved by a favorable genotype near the IL28B gene in HCV-infected patients [30]. These results prompt a possible pre-treatment prediction of therapy success or failure in a near future.

The identification and development of NS3/4A protease inhibitors has highlighted new therapeutic perspectives for the treatment of HCV chronic infection. In combination with other classes of DAA (NS5B polymerase, NS5A, entry or assembly inhibitors), these new treatments will hopefully be better tolerated and will cure HCV-infected patients.

## Figures and Tables

**Table 1.**
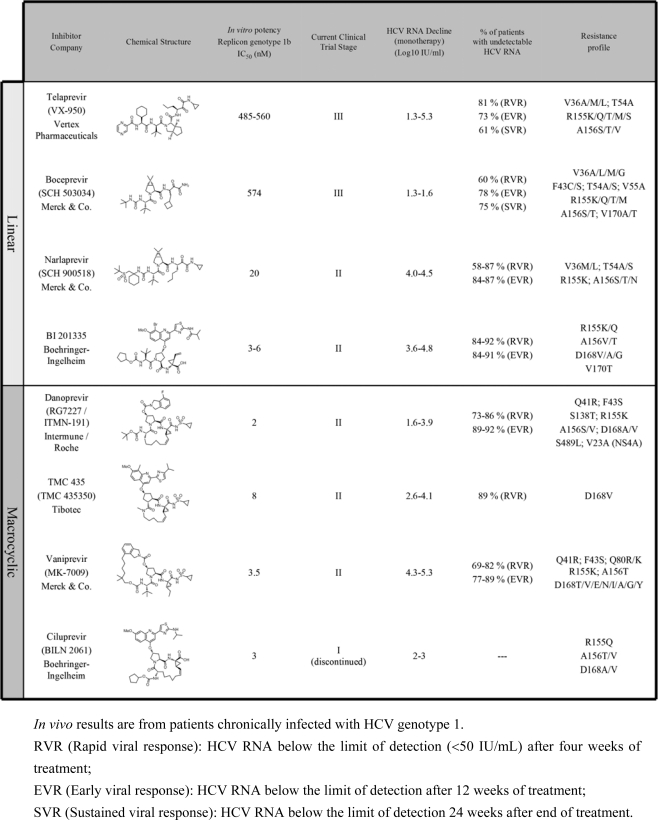
*In vivo* and *in vitro* characteristics and potency of HCV protease inhibitors currently in clinical development.
